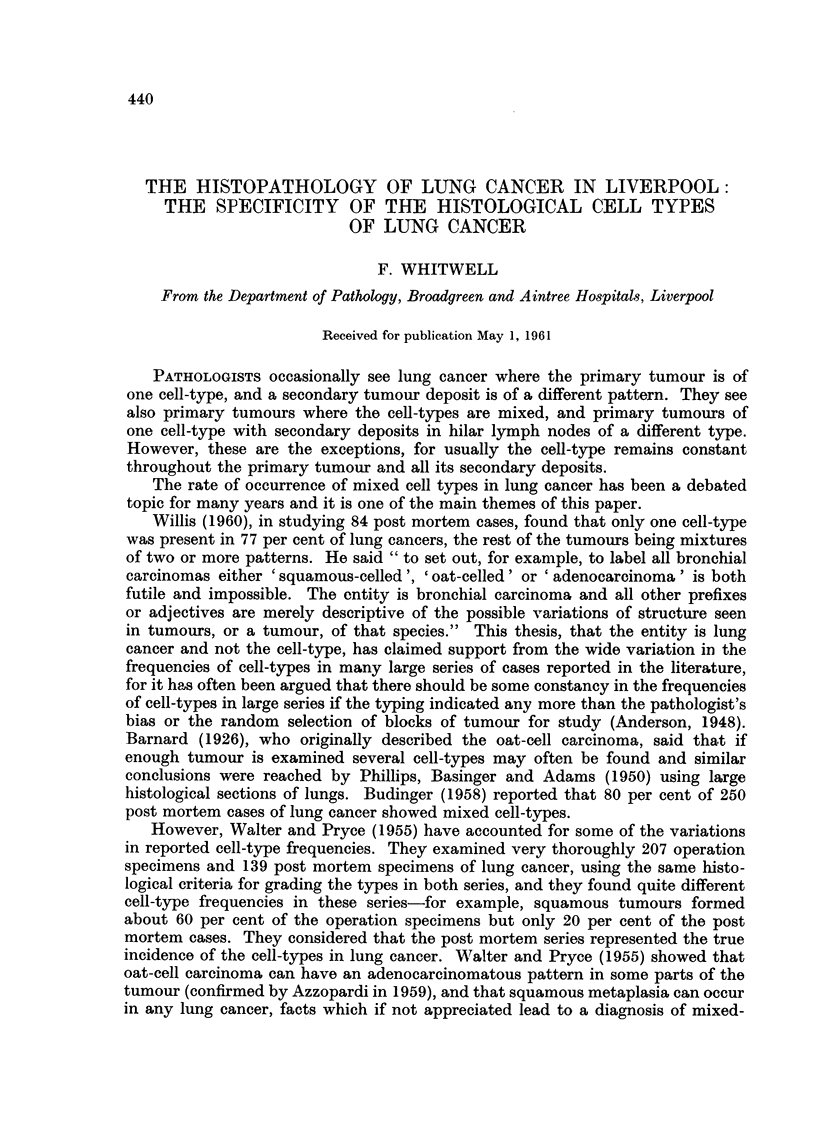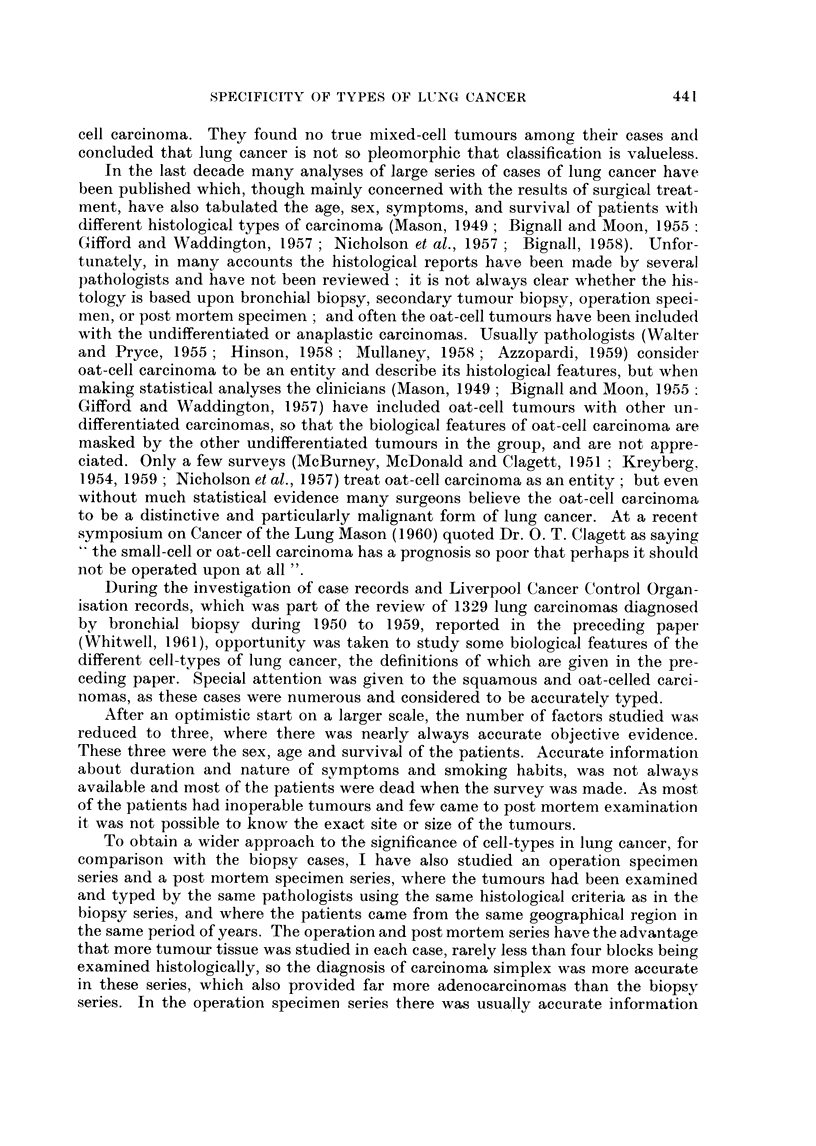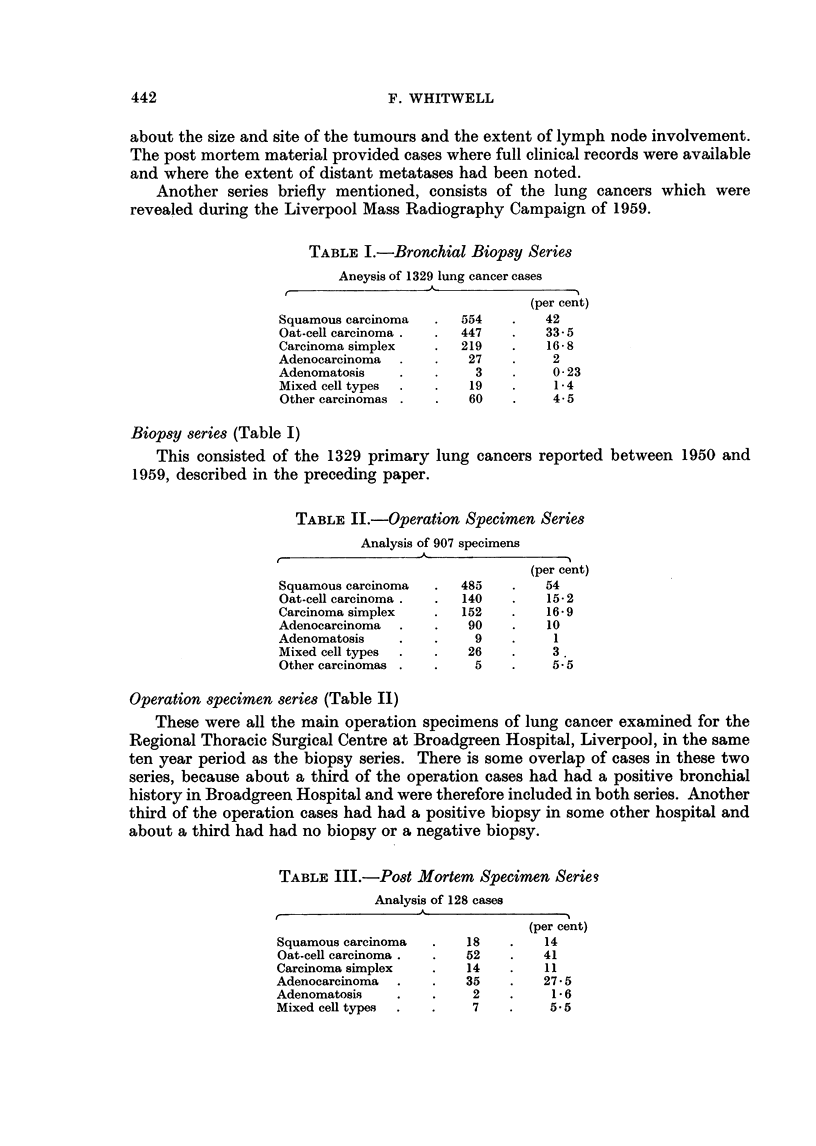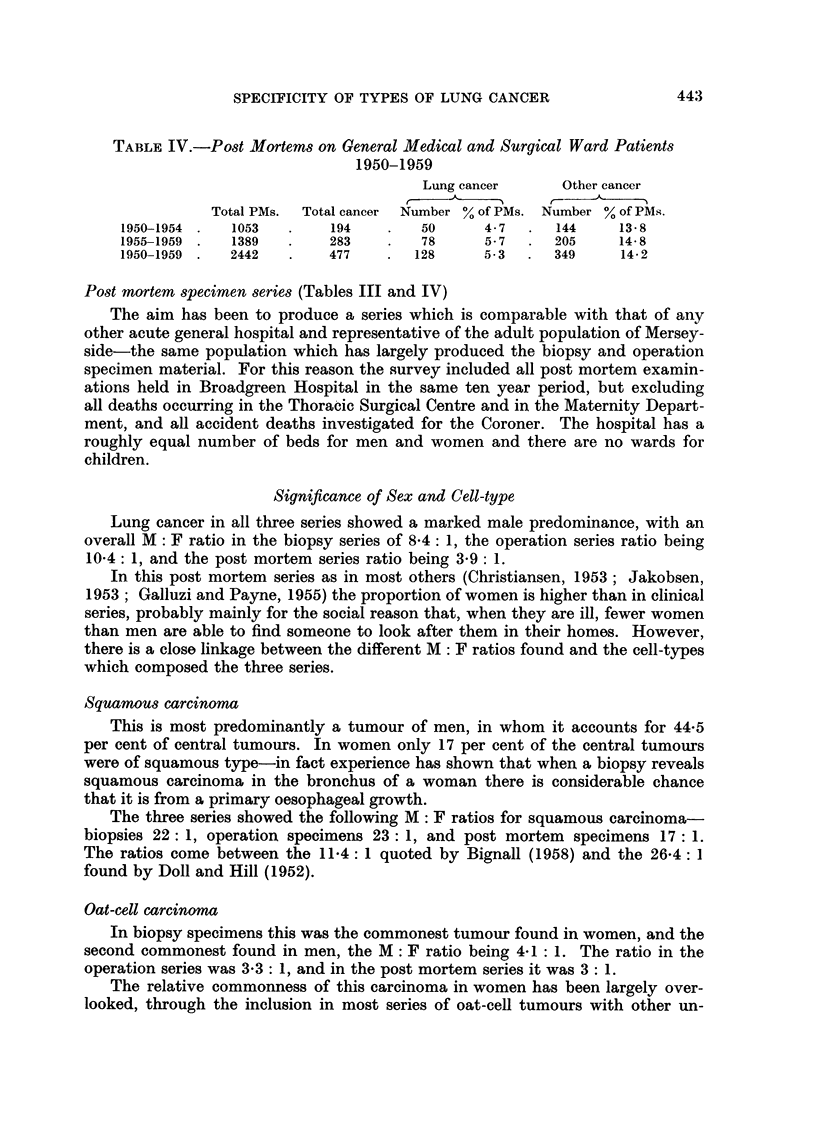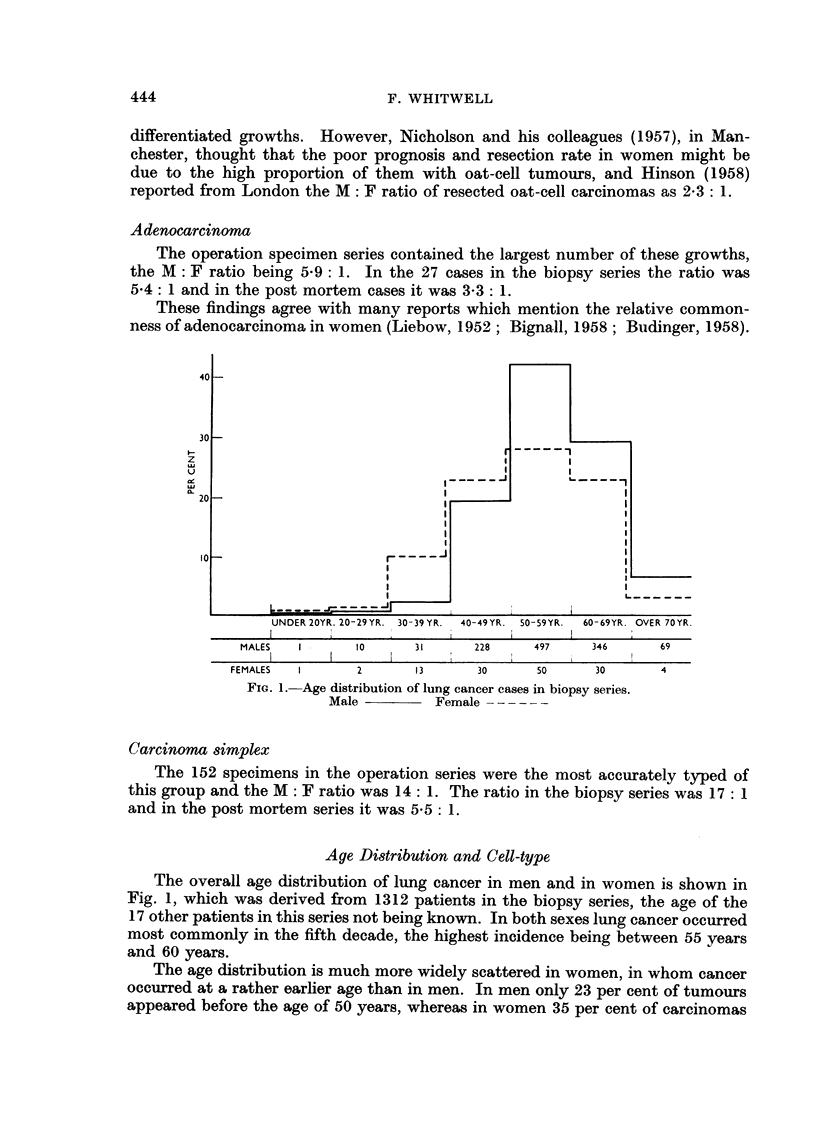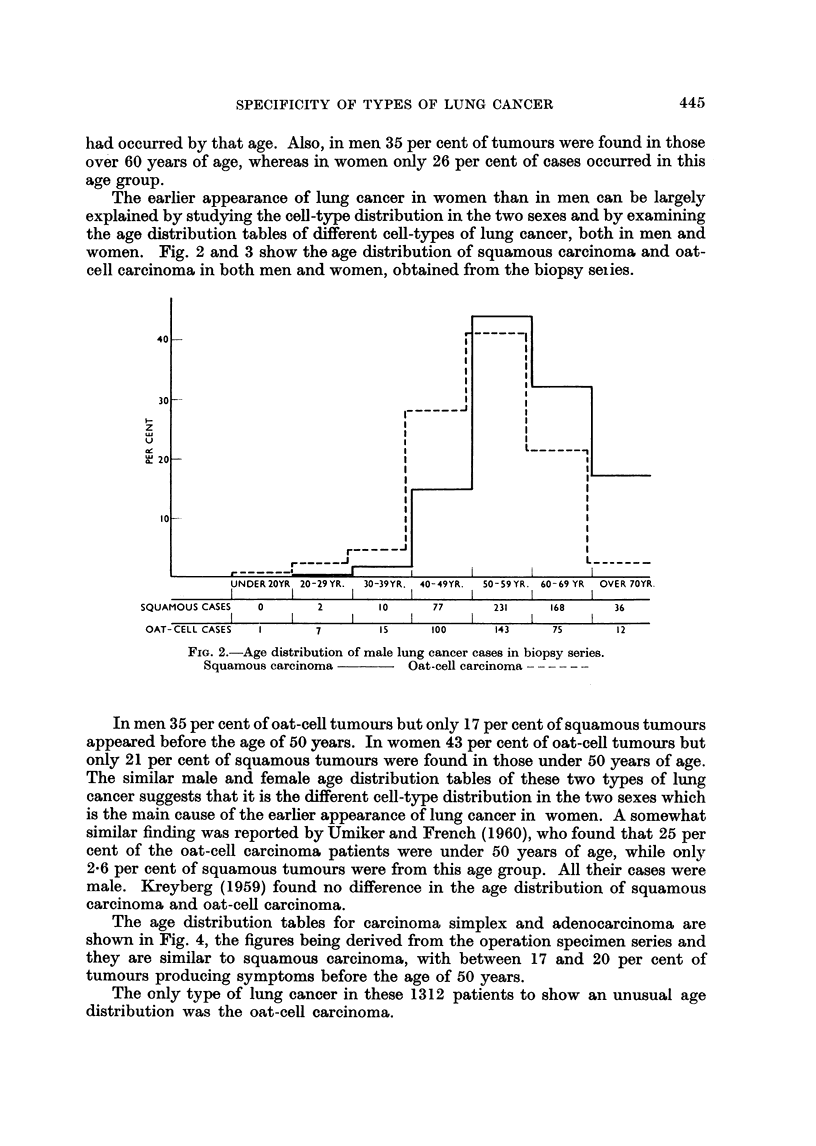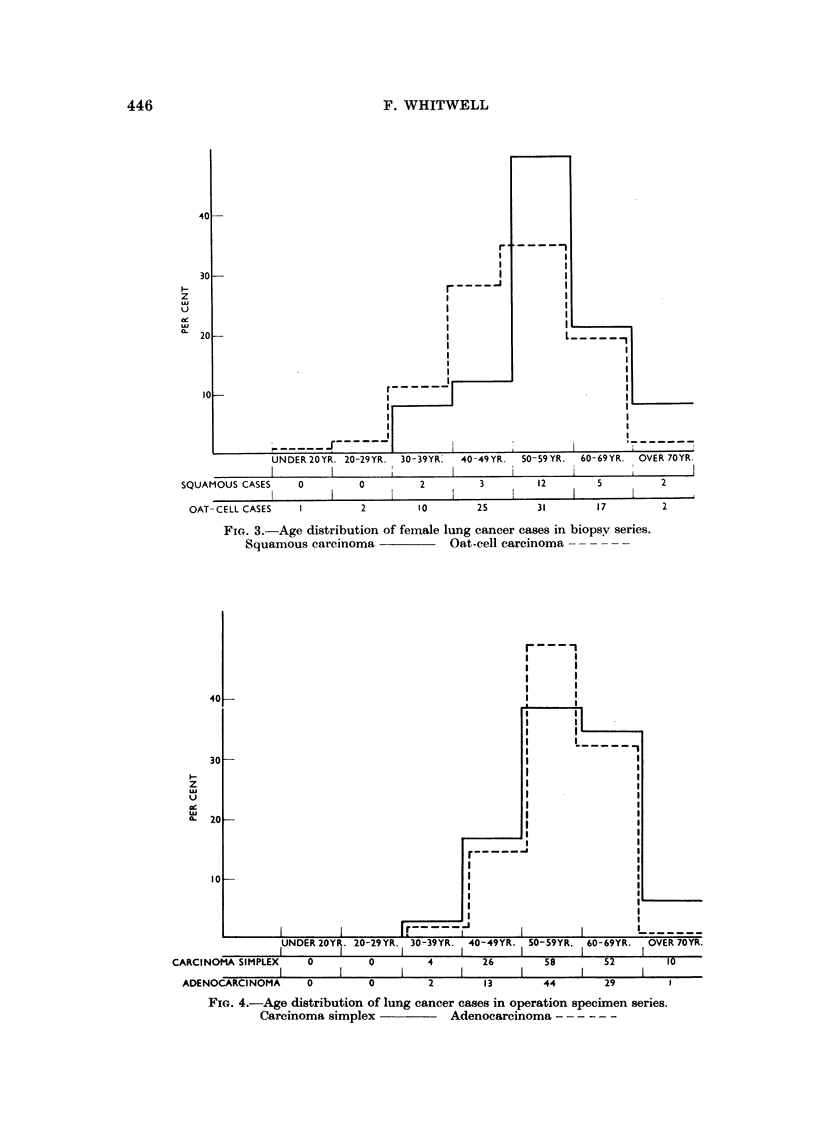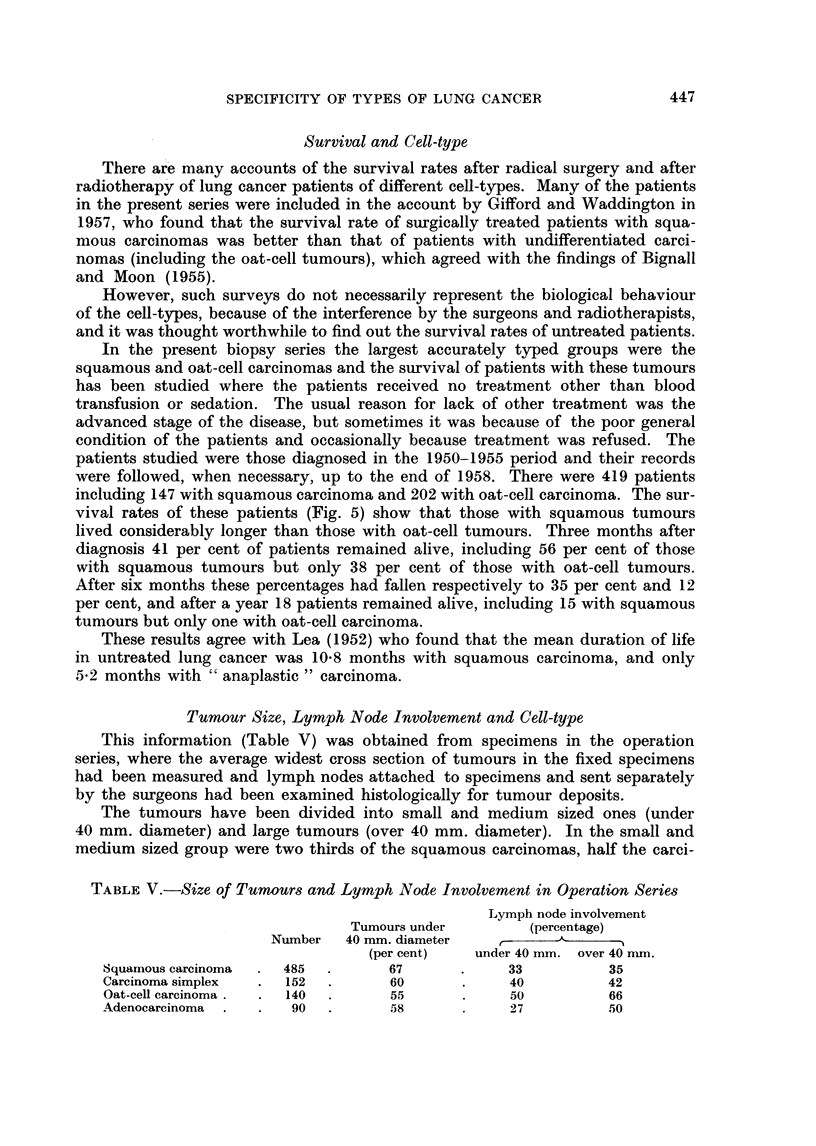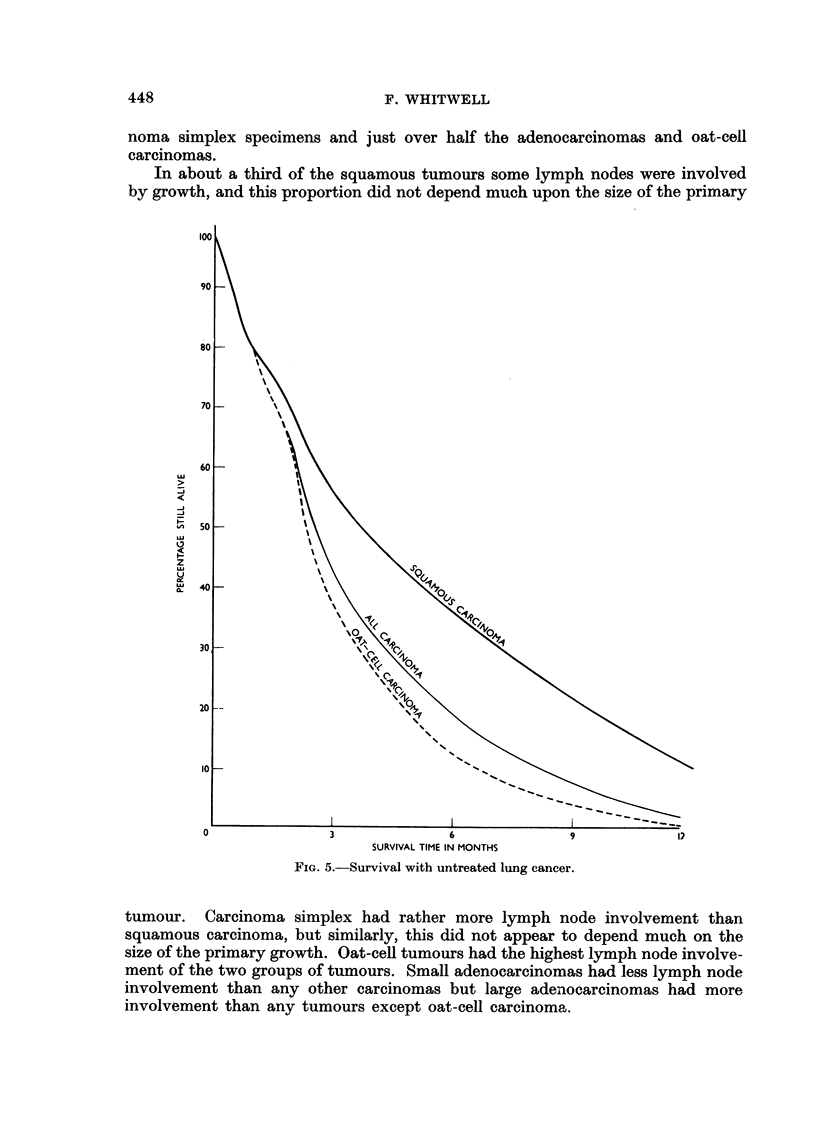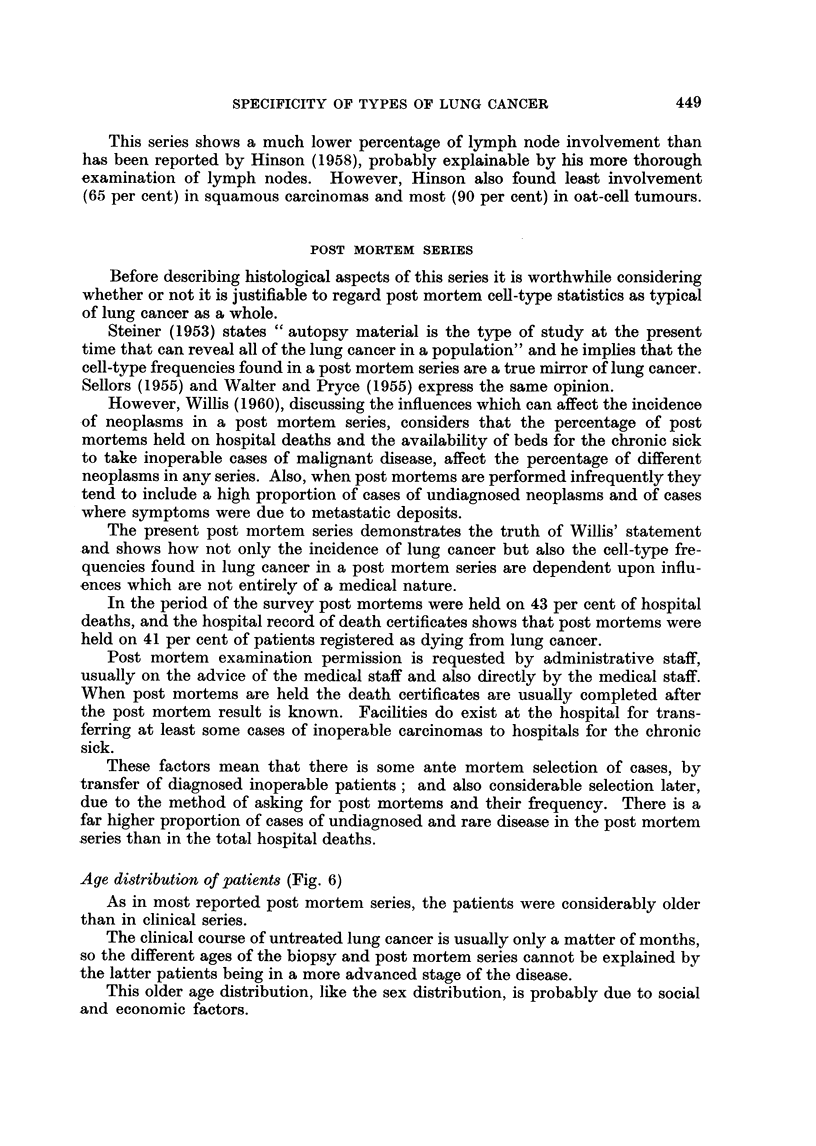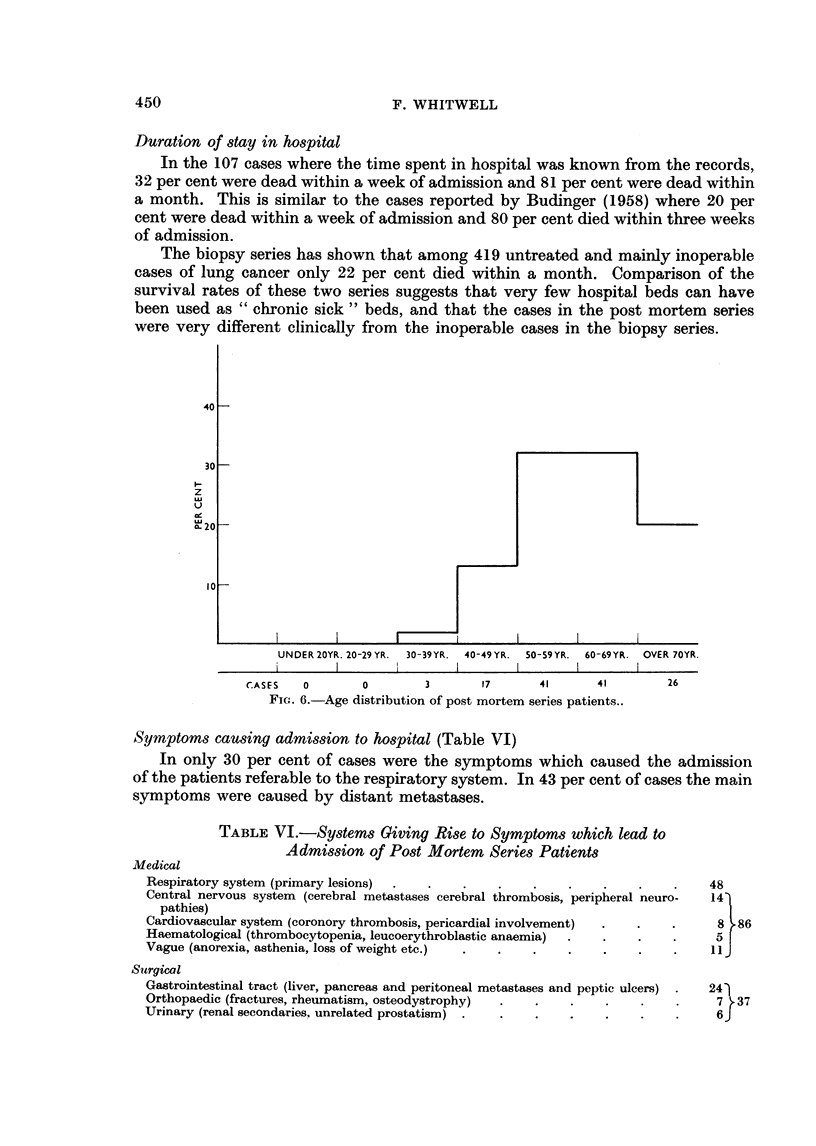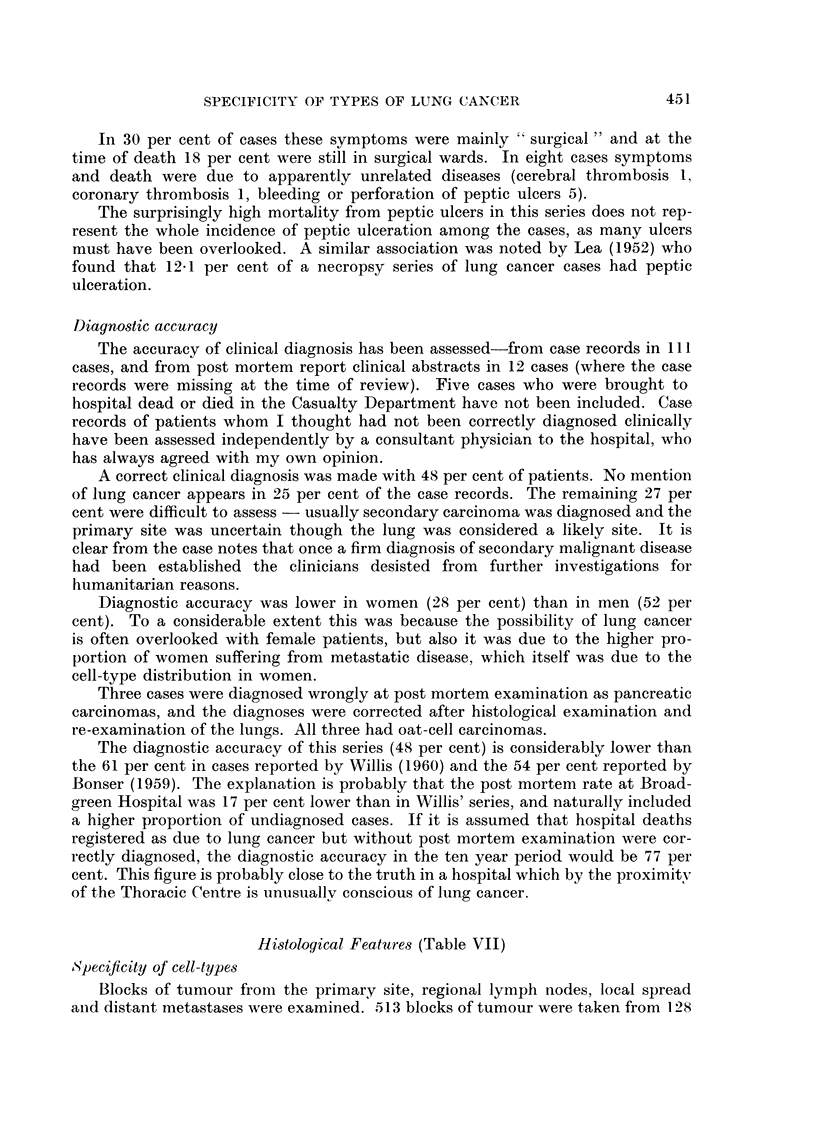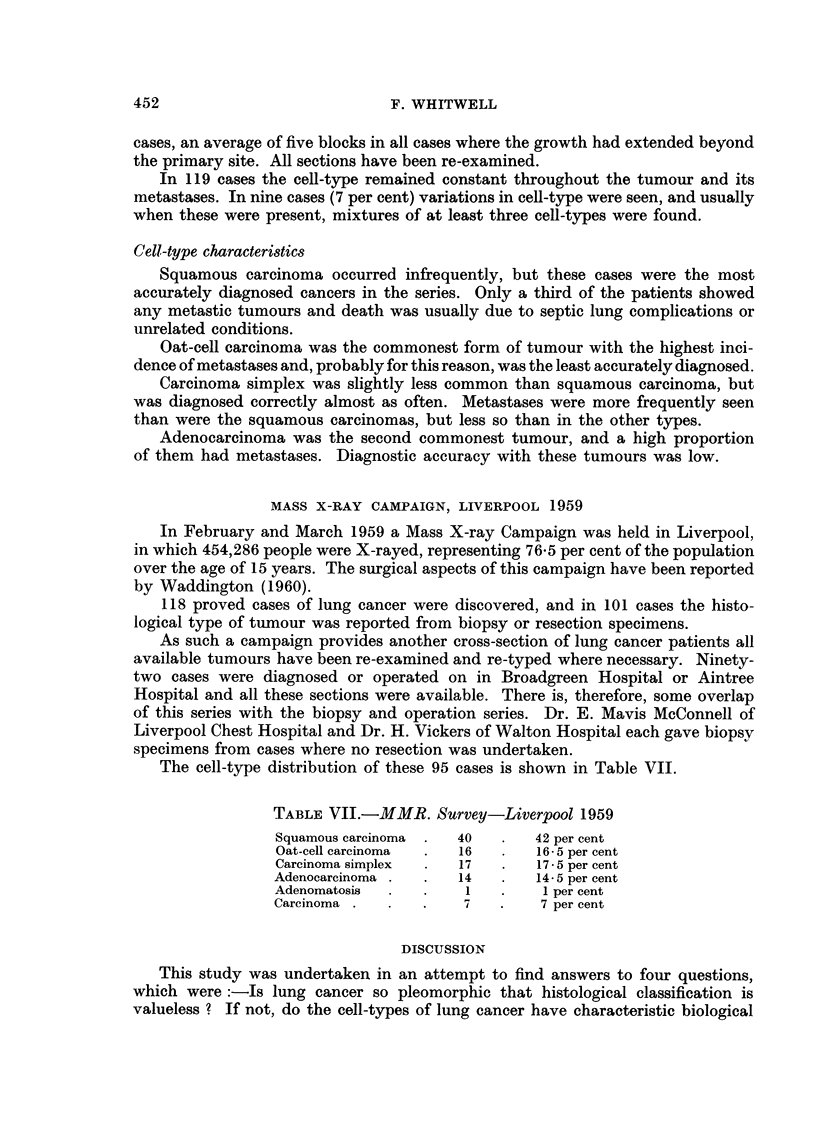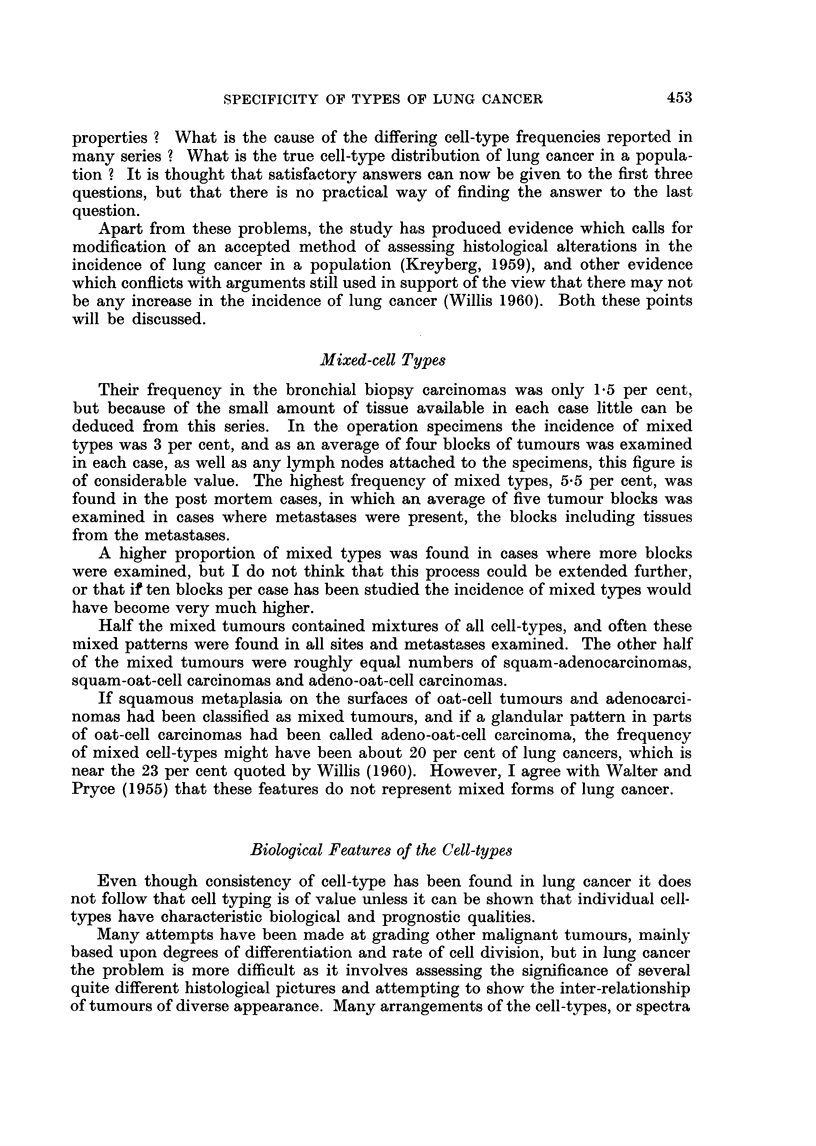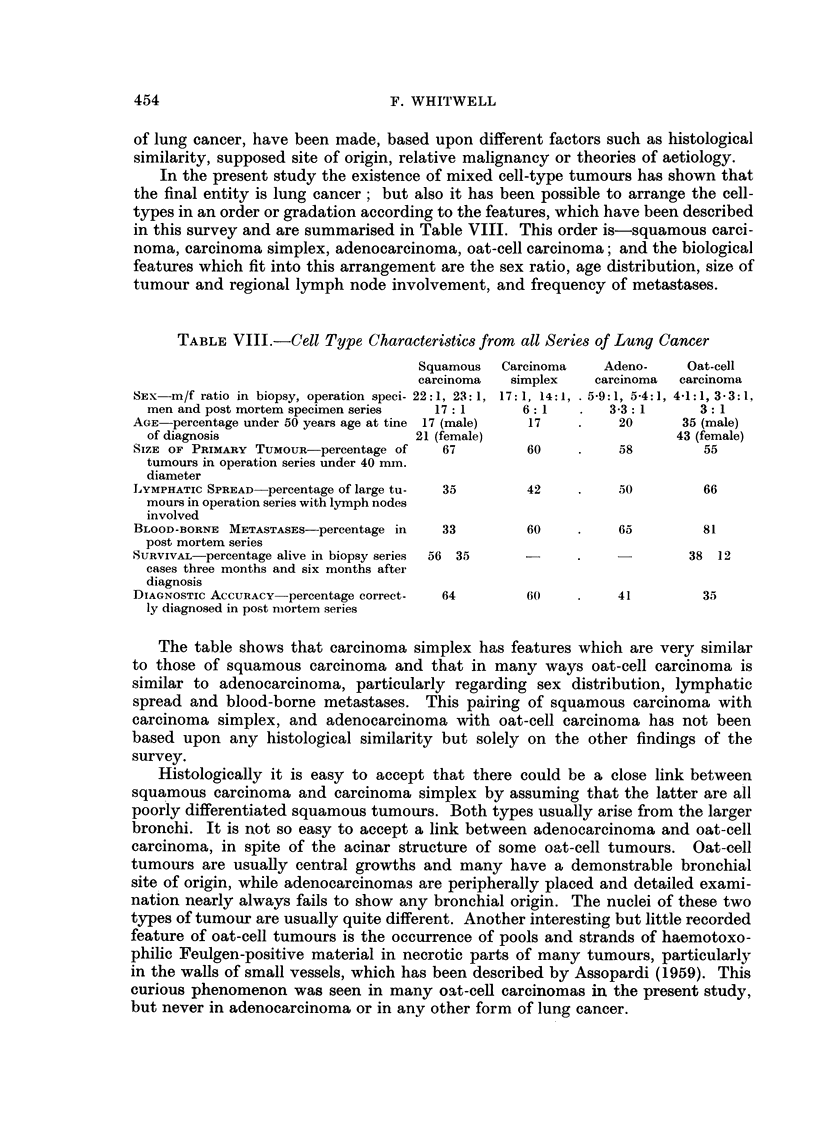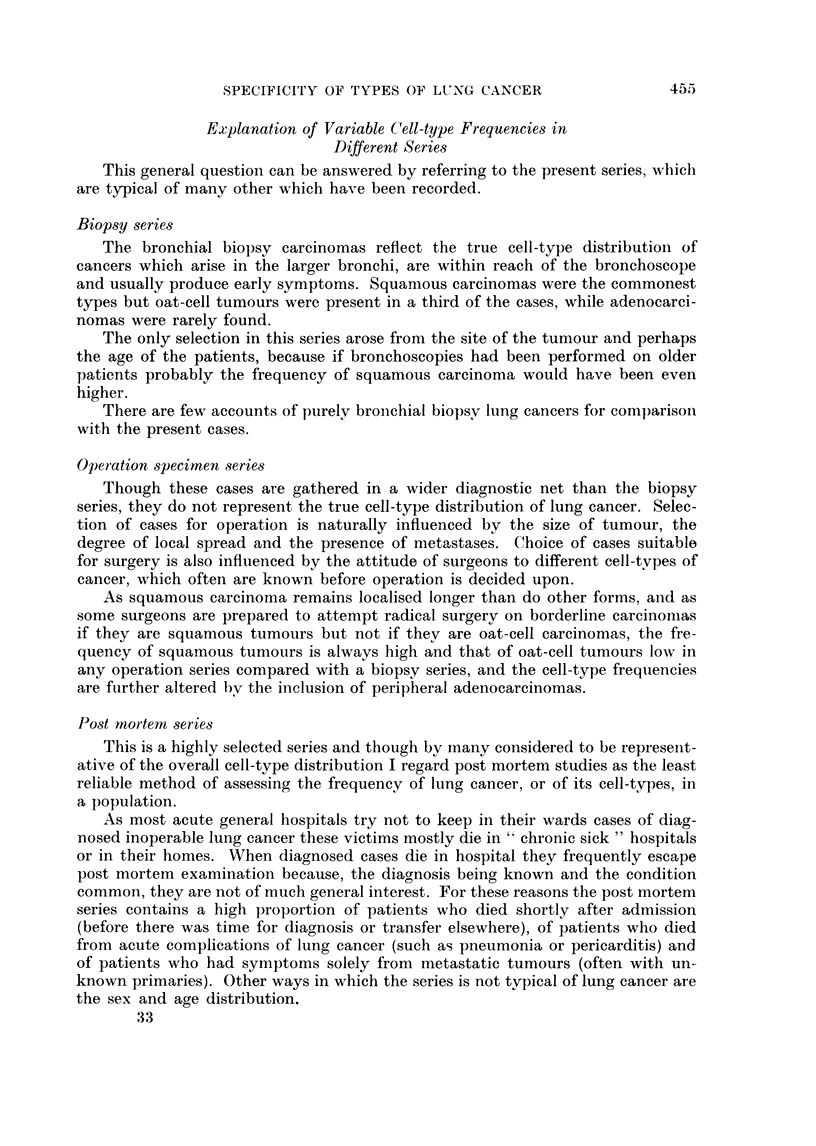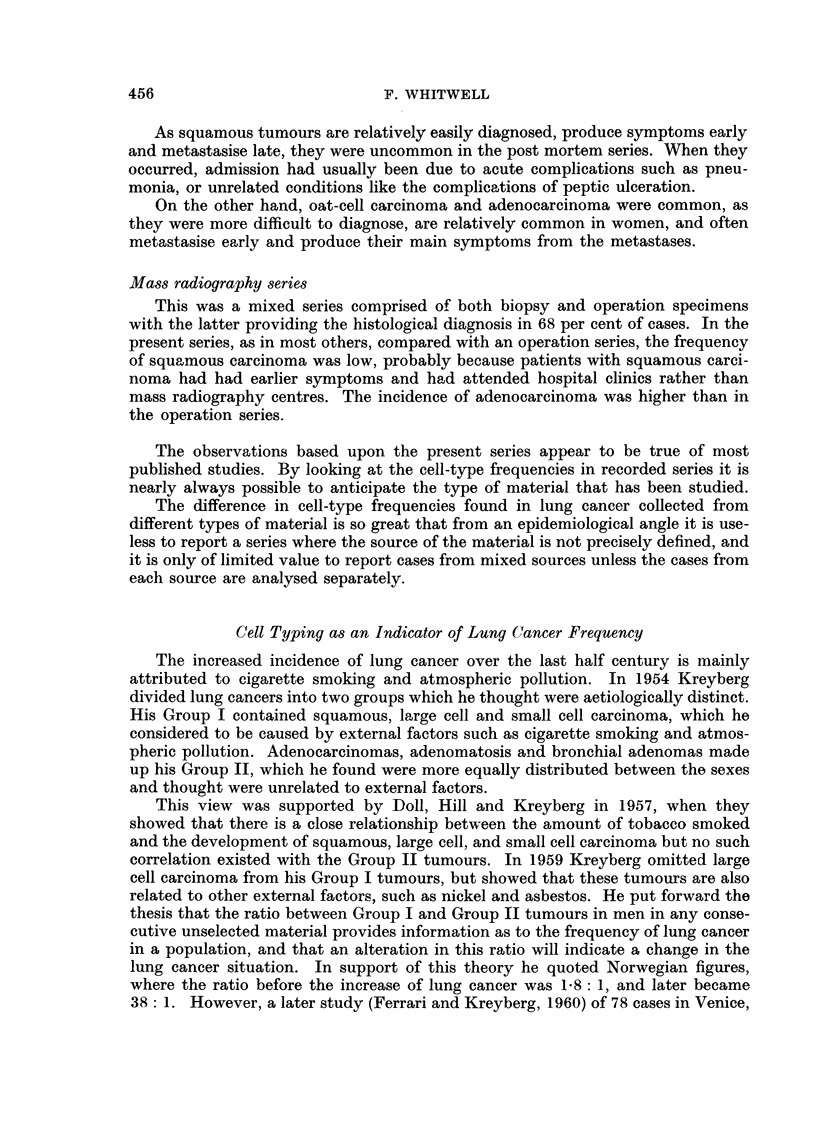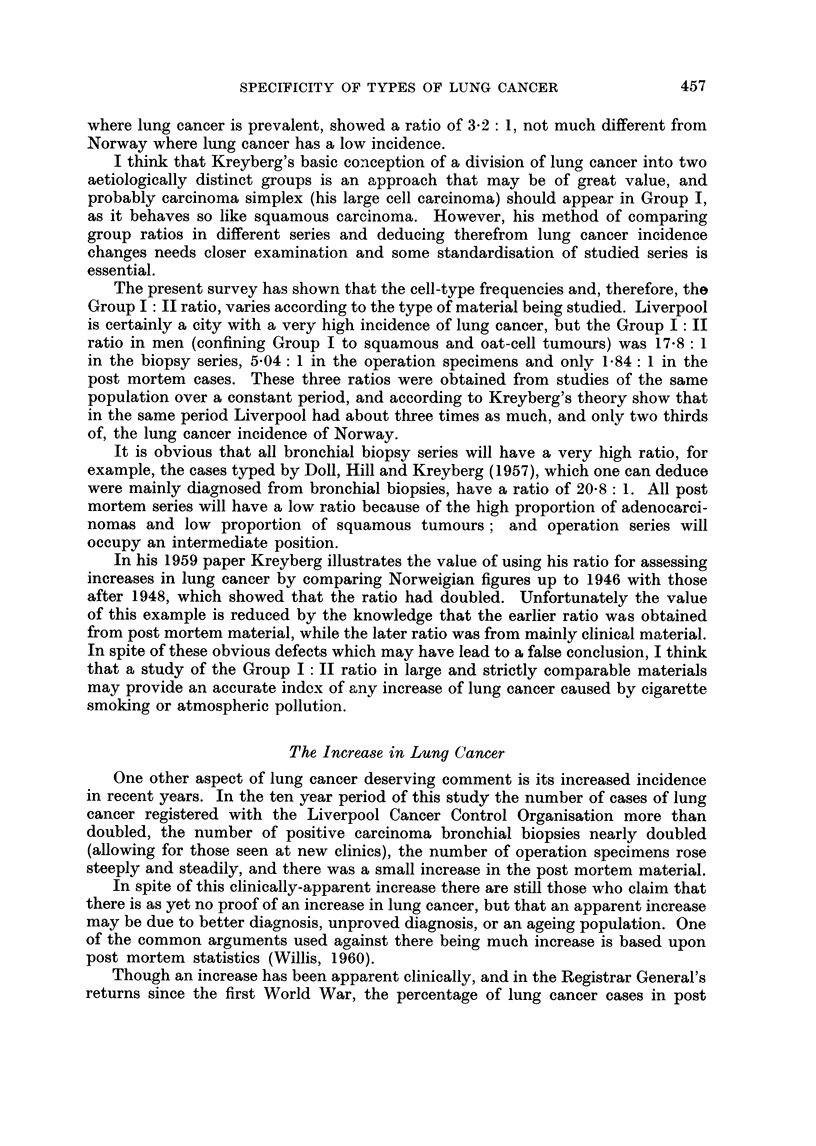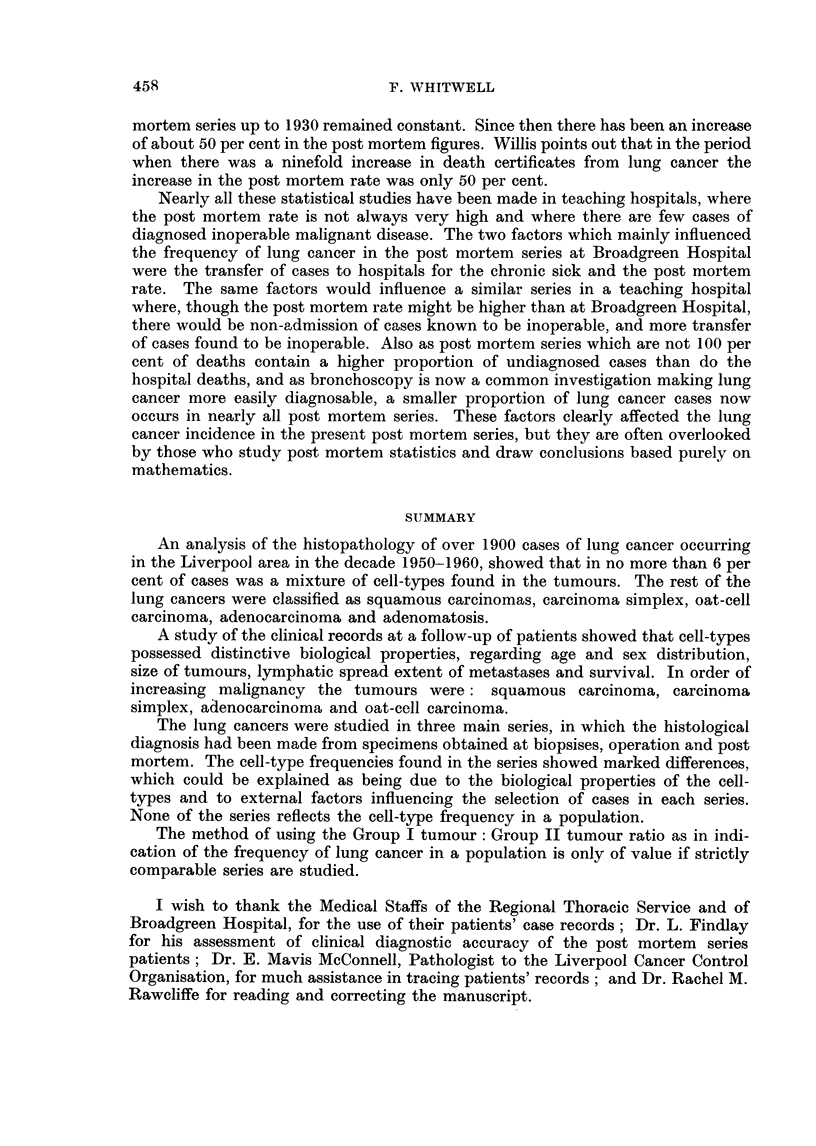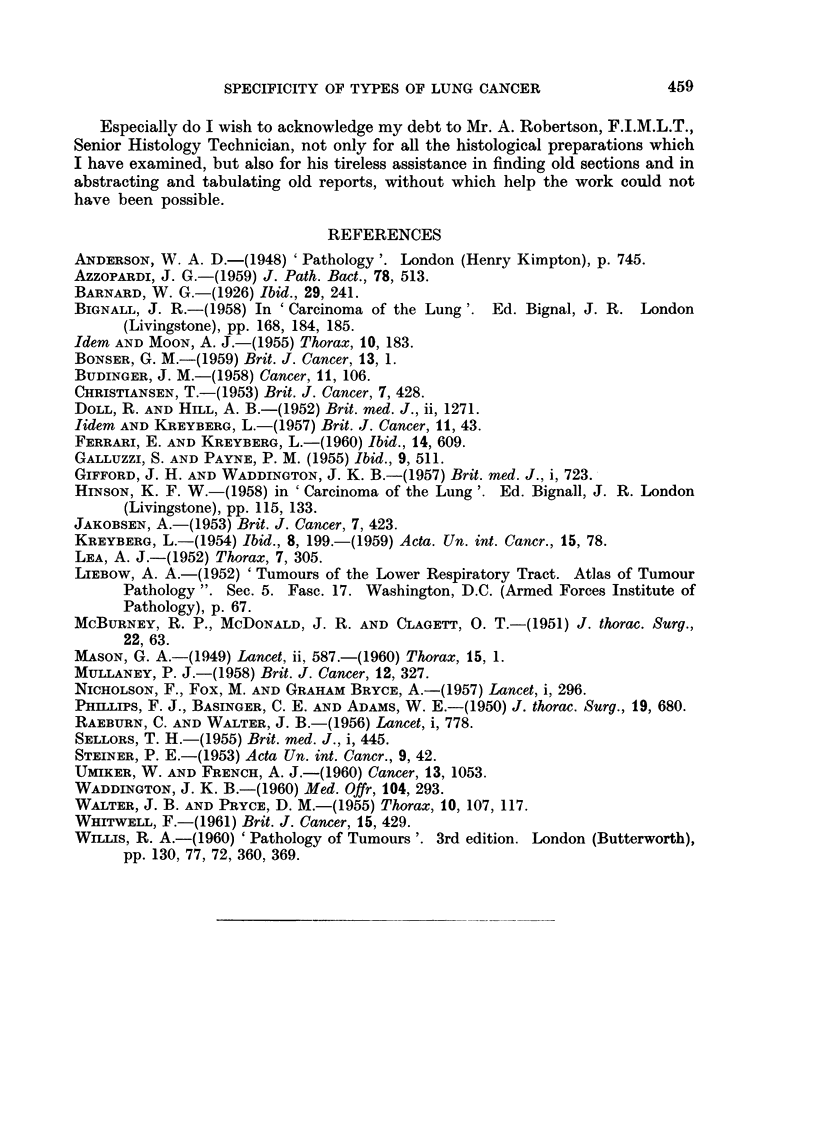# The Histopathology of Lung Cancer in Liverpool: The Specificity of the Histological Cell Types of Lung Cancer

**DOI:** 10.1038/bjc.1961.54

**Published:** 1961-09

**Authors:** F. Whitwell


### THE HISTOPATHOLOGY OF LUNG CANCER IN LIVERPOOL: THE SPECIFICITY OF THE HISTOLOGICAL CELL TYPES OF LUNG CANCER

F. WHITWELL

*From the Department of Pathology, Broadgreen and Aintree Hospitals, Liverpool*

Received for publication May 1, 1961

PATHOLOGISTS occasionally see lung cancer where the primary tumour is of one cell-type, and a secondary tumour deposit is of a different pattern. They see also primary tumours where the cell-types are mixed, and primary tumours of one cell-type with secondary deposits in hilar lymph nodes of a different type. However, these are the exceptions, for usually the cell-type remains constant throughout the primary tumour and all its secondary deposits.

The rate of occurrence of mixed cell types in lung cancer has been a debated topic for many years and it is one of the main themes of this paper.

Willis (1960), in studying 84 post mortem cases, found that only one cell-type was present in 77 per cent of lung cancers, the rest of the tumours being mixtures of two or more patterns. He said " to set out, for example, to label all bronchial carcinomas either 'squamous-celled', 'oat-celled' or 'adenocarcinoma' is both futile and impossible. The entity is bronchial carcinoma and all other prefixes or adjectives are merely descriptive of the possible variations of structure seen in tumours, or a tumour, of that species." This thesis, that the entity is lung cancer and not the cell-type, has claimed support from the wide variation in the frequencies of cell-types in many large series of cases reported in the literature, for it has often been argued that there should be some constancy in the frequencies of cell-types in large series if the typing indicated any more than the pathologist's bias or the random selection of blocks of tumour for study (Anderson, 1948). Barnard (1926), who originally described the oat-cell carcinoma, said that if enough tumour is examined several cell-types may often be found and similar conclusions were reached by Phillips, Basinger and Adams (1950) using large histological sections of lungs. Budinger (1958) reported that 80 per cent of 250 post mortem cases of lung cancer showed mixed cell-types.

However, Walter and Pryce (1955) have accounted for some of the variations in reported cell-type frequencies. They examined very thoroughly 207 operation specimens and 139 post mortem specimens of lung cancer, using the same histological criteria for grading the types in both series, and they found quite different cell-type frequencies in these series—for example, squamous tumours formed about 60 per cent of the operation specimens but only 20 per cent of the post mortem cases. They considered that the post mortem series represented the true incidence of the cell-types in lung cancer. Walter and Pryce (1955) showed that oat-cell carcinoma can have an adenocarcinomatous pattern in some parts of the tumour (confirmed by Azzopardi in 1959), and that squamous metaplasia can occur in any lung cancer, facts which if not appreciated lead to a diagnosis of mixedcell carcinoma. They found no true mixed-cell tumours among their cases and concluded that lung cancer is not so pleomorphic that classification is valueless.

In the last decade many analyses of large series of cases of lung cancer have been published which, though mainly concerned with the results of surgical treatment, have also tabulated the age, sex, symptoms, and survival of patients with different histological types of carcinoma (Mason, 1949 ; Bignall and Moon, 1955 ; Gifford and Waddington, 1957 ; Nicholson *et al.*, 1957 ; Bignall, 1958). Unfortunately, in many accounts the histological reports have been made by several pathologists and have not been reviewed ; it is not always clear whether the histology is based upon bronchial biopsy, secondary tumour biopsy, operation specimen, or post mortem specimen ; and often the oat-cell tumours have been included with the undifferentiated or anaplastic carcinomas. Usually pathologists (Walter and Pryce, 1955 ; Hinson, 1958 ; Mullaney, 1958 ; Azzopardi, 1959) consider oat-cell carcinoma to be an entity and describe its histological features, but when making statistical analyses the clinicians (Mason, 1949 ; Bignall and Moon, 1955 ; Gifford and Waddington, 1957) have included oat-cell tumours with other undifferentiated carcinomas, so that the biological features of oat-cell carcinoma are masked by the other undifferentiated tumours in the group, and are not appreciated. Only a few surveys (McBurney, McDonald and Clagett, 1951 ; Kreyberg, 1954, 1959 ; Nicholson *et al.*, 1957) treat oat-cell carcinoma as an entity ; but even without much statistical evidence many surgeons believe the oat-cell carcinoma to be a distinctive and particularly malignant form of lung cancer. At a recent symposium on Cancer of the Lung Mason (1960) quoted Dr. O. T. Clagett as saying " the small-cell or oat-cell carcinoma has a prognosis so poor that perhaps it should not be operated upon at all ".

During the investigation of case records and Liverpool Cancer Control Organisation records, which was part of the review of 1329 lung carcinomas diagnosed by bronchial biopsy during 1950 to 1959, reported in the preceding paper (Whitwell, 1961), opportunity was taken to study some biological features of the different cell-types of lung cancer, the definitions of which are given in the preceding paper. Special attention was given to the squamous and oat-celled carcinomas, as these cases were numerous and considered to be accurately typed.

After an optimistic start on a larger scale, the number of factors studied was reduced to three, where there was nearly always accurate objective evidence. These three were the sex, age and survival of the patients. Accurate information about duration and nature of symptoms and smoking habits, was not always available and most of the patients were dead when the survey was made. As most of the patients had inoperable tumours and few came to post mortem examination it was not possible to know the exact site or size of the tumours.

To obtain a wider approach to the significance of cell-types in lung cancer, for comparison with the biopsy cases, I have also studied an operation specimen series and a post mortem specimen series, where the tumours had been examined and typed by the same pathologists using the same histological criteria as in the biopsy series, and where the patients came from the same geographical region in the same period of years. The operation and post mortem series have the advantage that more tumour tissue was studied in each case, rarely less than four blocks being examined histologically, so the diagnosis of carcinoma simplex was more accurate in these series, which also provided far more adenocarcinomas than the biopsy series. In the operation specimen series there was usually accurate information about the size and site of the tumours and the extent of lymph node involvement. The post mortem material provided cases where full clinical records were available and where the extent of distant metatases had been noted.

Another series briefly mentioned, consists of the lung cancers which were revealed during the Liverpool Mass Radiography Campaign of 1959.

TABLE I.—*Bronchial Biopsy Series*

| | Aneysis of 1329 lung cancer cases | |
|---|---|---|
| | | (per cent) |
| Squamous carcinoma | 554 | 42 |
| Oat-cell carcinoma | 447 | 33·5 |
| Carcinoma simplex | 219 | 16·8 |
| Adenocarcinoma | 27 | 2 |
| Adenomatosis | 3 | 0·23 |
| Mixed cell types | 19 | 1·4 |
| Other carcinomas | 60 | 4·5 |

*Biopsy series* (Table I)

This consisted of the 1329 primary lung cancers reported between 1950 and 1959, described in the preceding paper.

TABLE II.—*Operation Specimen Series*

| | Analysis of 907 specimens | |
|---|---|---|
| | | (per cent) |
| Squamous carcinoma | 485 | 54 |
| Oat-cell carcinoma | 140 | 15·2 |
| Carcinoma simplex | 152 | 16·9 |
| Adenocarcinoma | 90 | 10 |
| Adenomatosis | 9 | 1 |
| Mixed cell types | 26 | 3 |
| Other carcinomas | 5 | 5·5 |

*Operation specimen series* (Table II)

These were all the main operation specimens of lung cancer examined for the Regional Thoracic Surgical Centre at Broadgreen Hospital, Liverpool, in the same ten year period as the biopsy series. There is some overlap of cases in these two series, because about a third of the operation cases had had a positive bronchial history in Broadgreen Hospital and were therefore included in both series. Another third of the operation cases had had a positive biopsy in some other hospital and about a third had had no biopsy or a negative biopsy.

TABLE III.—*Post Mortem Specimen Series*

| | Analysis of 128 cases | |
|---|---|---|
| | | (per cent) |
| Squamous carcinoma | 18 | 14 |
| Oat-cell carcinoma | 52 | 41 |
| Carcinoma simplex | 14 | 11 |
| Adenocarcinoma | 35 | 27·5 |
| Adenomatosis | 2 | 1·6 |
| Mixed cell types | 7 | 5·5 |

TABLE IV.—*Post Mortems on General Medical and Surgical Ward Patients* 1950–1959

| | Total PMs. | Total cancer | Lung cancer Number | Lung cancer % of PMs. | Other cancer Number | Other cancer % of PMs. |
|---|---|---|---|---|---|---|
| 1950–1954 | 1053 | 194 | 50 | 4·7 | 144 | 13·8 |
| 1955–1959 | 1389 | 283 | 78 | 5·7 | 205 | 14·8 |
| 1950–1959 | 2442 | 477 | 128 | 5·3 | 349 | 14·2 |

*Post mortem specimen series* (Tables III and IV)

The aim has been to produce a series which is comparable with that of any other acute general hospital and representative of the adult population of Merseyside—the same population which has largely produced the biopsy and operation specimen material. For this reason the survey included all post mortem examinations held in Broadgreen Hospital in the same ten year period, but excluding all deaths occurring in the Thoracic Surgical Centre and in the Maternity Department, and all accident deaths investigated for the Coroner. The hospital has a roughly equal number of beds for men and women and there are no wards for children.

*Significance of Sex and Cell-type*

Lung cancer in all three series showed a marked male predominance, with an overall M : F ratio in the biopsy series of 8·4 : 1, the operation series ratio being 10·4 : 1, and the post mortem series ratio being 3·9 : 1.

In this post mortem series as in most others (Christiansen, 1953 ; Jakobsen, 1953 ; Galluzi and Payne, 1955) the proportion of women is higher than in clinical series, probably mainly for the social reason that, when they are ill, fewer women than men are able to find someone to look after them in their homes. However, there is a close linkage between the different M : F ratios found and the cell-types which composed the three series.

*Squamous carcinoma*

This is most predominantly a tumour of men, in whom it accounts for 44·5 per cent of central tumours. In women only 17 per cent of the central tumours were of squamous type—in fact experience has shown that when a biopsy reveals squamous carcinoma in the bronchus of a woman there is considerable chance that it is from a primary oesophageal growth.

The three series showed the following M : F ratios for squamous carcinoma—biopsies 22 : 1, operation specimens 23 : 1, and post mortem specimens 17 : 1. The ratios come between the 11·4 : 1 quoted by Bignall (1958) and the 26·4 : 1 found by Doll and Hill (1952).

*Oat-cell carcinoma*

In biopsy specimens this was the commonest tumour found in women, and the second commonest found in men, the M : F ratio being 4·1 : 1. The ratio in the operation series was 3·3 : 1, and in the post mortem series it was 3 : 1.

The relative commonness of this carcinoma in women has been largely overlooked, through the inclusion in most series of oat-cell tumours with other undifferentiated growths. However, Nicholson and his colleagues (1957), in Manchester, thought that the poor prognosis and resection rate in women might be due to the high proportion of them with oat-cell tumours, and Hinson (1958) reported from London the M : F ratio of resected oat-cell carcinomas as 2·3 : 1.

*Adenocarcinoma*

The operation specimen series contained the largest number of these growths, the M : F ratio being 5·9 : 1. In the 27 cases in the biopsy series the ratio was 5·4 : 1 and in the post mortem cases it was 3·3 : 1.

These findings agree with many reports which mention the relative commonness of adenocarcinoma in women (Liebow, 1952 ; Bignall, 1958 ; Budinger, 1958).

| | UNDER 20YR. | 20-29YR. | 30-39YR. | 40-49YR. | 50-59YR. | 60-69YR. | OVER 70YR. |
|---|---|---|---|---|---|---|---|
| MALES | 1 | 10 | 31 | 228 | 497 | 346 | 69 |
| FEMALES | 1 | 2 | 13 | 30 | 50 | 30 | 4 |

FIG. 1.—Age distribution of lung cancer cases in biopsy series.
Male ———— Female – – – – – –

*Carcinoma simplex*

The 152 specimens in the operation series were the most accurately typed of this group and the M : F ratio was 14 : 1. The ratio in the biopsy series was 17 : 1 and in the post mortem series it was 5·5 : 1.

### *Age Distribution and Cell-type*

The overall age distribution of lung cancer in men and in women is shown in Fig. 1, which was derived from 1312 patients in the biopsy series, the age of the 17 other patients in this series not being known. In both sexes lung cancer occurred most commonly in the fifth decade, the highest incidence being between 55 years and 60 years.

The age distribution is much more widely scattered in women, in whom cancer occurred at a rather earlier age than in men. In men only 23 per cent of tumours appeared before the age of 50 years, whereas in women 35 per cent of carcinomas had occurred by that age. Also, in men 35 per cent of tumours were found in those over 60 years of age, whereas in women only 26 per cent of cases occurred in this age group.

The earlier appearance of lung cancer in women than in men can be largely explained by studying the cell-type distribution in the two sexes and by examining the age distribution tables of different cell-types of lung cancer, both in men and women. Fig. 2 and 3 show the age distribution of squamous carcinoma and oat-cell carcinoma in both men and women, obtained from the biopsy seiies.

| | UNDER 20YR | 20-29 YR. | 30-39YR. | 40-49YR. | 50-59 YR. | 60-69 YR | OVER 70YR. |
|---|---|---|---|---|---|---|---|
| SQUAMOUS CASES | 0 | 2 | 10 | 77 | 231 | 168 | 36 |
| OAT-CELL CASES | 1 | 7 | 15 | 100 | 143 | 75 | 12 |

Fig. 2.—Age distribution of male lung cancer cases in biopsy series.
Squamous carcinoma ———— Oat-cell carcinoma – – – – – –

In men 35 per cent of oat-cell tumours but only 17 per cent of squamous tumours appeared before the age of 50 years. In women 43 per cent of oat-cell tumours but only 21 per cent of squamous tumours were found in those under 50 years of age. The similar male and female age distribution tables of these two types of lung cancer suggests that it is the different cell-type distribution in the two sexes which is the main cause of the earlier appearance of lung cancer in women. A somewhat similar finding was reported by Umiker and French (1960), who found that 25 per cent of the oat-cell carcinoma patients were under 50 years of age, while only 2·6 per cent of squamous tumours were from this age group. All their cases were male. Kreyberg (1959) found no difference in the age distribution of squamous carcinoma and oat-cell carcinoma.

The age distribution tables for carcinoma simplex and adenocarcinoma are shown in Fig. 4, the figures being derived from the operation specimen series and they are similar to squamous carcinoma, with between 17 and 20 per cent of tumours producing symptoms before the age of 50 years.

The only type of lung cancer in these 1312 patients to show an unusual age distribution was the oat-cell carcinoma.

| | UNDER 20 YR. | 20-29 YR. | 30-39 YR. | 40-49 YR. | 50-59 YR. | 60-69 YR. | OVER 70 YR. |
|---|---|---|---|---|---|---|---|
| SQUAMOUS CASES | 0 | 0 | 2 | 3 | 12 | 5 | 2 |
| OAT-CELL CASES | 1 | 2 | 10 | 25 | 31 | 17 | 2 |

FIG. 3.—Age distribution of female lung cancer cases in biopsy series.
Squamous carcinoma ———— Oat-cell carcinoma – – – – – –

| | UNDER 20 YR. | 20-29 YR. | 30-39 YR. | 40-49 YR. | 50-59 YR. | 60-69 YR. | OVER 70 YR. |
|---|---|---|---|---|---|---|---|
| CARCINOMA SIMPLEX | 0 | 0 | 4 | 26 | 58 | 52 | 10 |
| ADENOCARCINOMA | 0 | 0 | 2 | 13 | 44 | 29 | 1 |

FIG. 4.—Age distribution of lung cancer cases in operation specimen series.
Carcinoma simplex ———— Adenocarcinoma – – – – – –

### *Survival and Cell-type*

There are many accounts of the survival rates after radical surgery and after radiotherapy of lung cancer patients of different cell-types. Many of the patients in the present series were included in the account by Gifford and Waddington in 1957, who found that the survival rate of surgically treated patients with squamous carcinomas was better than that of patients with undifferentiated carcinomas (including the oat-cell tumours), which agreed with the findings of Bignall and Moon (1955).

However, such surveys do not necessarily represent the biological behaviour of the cell-types, because of the interference by the surgeons and radiotherapists, and it was thought worthwhile to find out the survival rates of untreated patients.

In the present biopsy series the largest accurately typed groups were the squamous and oat-cell carcinomas and the survival of patients with these tumours has been studied where the patients received no treatment other than blood transfusion or sedation. The usual reason for lack of other treatment was the advanced stage of the disease, but sometimes it was because of the poor general condition of the patients and occasionally because treatment was refused. The patients studied were those diagnosed in the 1950–1955 period and their records were followed, when necessary, up to the end of 1958. There were 419 patients including 147 with squamous carcinoma and 202 with oat-cell carcinoma. The survival rates of these patients (Fig. 5) show that those with squamous tumours lived considerably longer than those with oat-cell tumours. Three months after diagnosis 41 per cent of patients remained alive, including 56 per cent of those with squamous tumours but only 38 per cent of those with oat-cell tumours. After six months these percentages had fallen respectively to 35 per cent and 12 per cent, and after a year 18 patients remained alive, including 15 with squamous tumours but only one with oat-cell carcinoma.

These results agree with Lea (1952) who found that the mean duration of life in untreated lung cancer was 10·8 months with squamous carcinoma, and only 5·2 months with " anaplastic " carcinoma.

### *Tumour Size, Lymph Node Involvement and Cell-type*

This information (Table V) was obtained from specimens in the operation series, where the average widest cross section of tumours in the fixed specimens had been measured and lymph nodes attached to specimens and sent separately by the surgeons had been examined histologically for tumour deposits.

The tumours have been divided into small and medium sized ones (under 40 mm. diameter) and large tumours (over 40 mm. diameter). In the small and medium sized group were two thirds of the squamous carcinomas, half the carci-

TABLE V.—*Size of Tumours and Lymph Node Involvement in Operation Series*

| | Number | Tumours under 40 mm. diameter (per cent) | Lymph node involvement (percentage) | |
|---|---|---|---|---|
| | | | under 40 mm. | over 40 mm. |
| Squamous carcinoma | 485 | 67 | 33 | 35 |
| Carcinoma simplex | 152 | 60 | 40 | 42 |
| Oat-cell carcinoma | 140 | 55 | 50 | 66 |
| Adenocarcinoma | 90 | 58 | 27 | 50 |

noma simplex specimens and just over half the adenocarcinomas and oat-cell carcinomas.

In about a third of the squamous tumours some lymph nodes were involved by growth, and this proportion did not depend much upon the size of the primary

FIG. 5.—Survival with untreated lung cancer.

tumour. Carcinoma simplex had rather more lymph node involvement than squamous carcinoma, but similarly, this did not appear to depend much on the size of the primary growth. Oat-cell tumours had the highest lymph node involvement of the two groups of tumours. Small adenocarcinomas had less lymph node involvement than any other carcinomas but large adenocarcinomas had more involvement than any tumours except oat-cell carcinoma.

This series shows a much lower percentage of lymph node involvement than has been reported by Hinson (1958), probably explainable by his more thorough examination of lymph nodes. However, Hinson also found least involvement (65 per cent) in squamous carcinomas and most (90 per cent) in oat-cell tumours.

### POST MORTEM SERIES

Before describing histological aspects of this series it is worthwhile considering whether or not it is justifiable to regard post mortem cell-type statistics as typical of lung cancer as a whole.

Steiner (1953) states " autopsy material is the type of study at the present time that can reveal all of the lung cancer in a population" and he implies that the cell-type frequencies found in a post mortem series are a true mirror of lung cancer. Sellors (1955) and Walter and Pryce (1955) express the same opinion.

However, Willis (1960), discussing the influences which can affect the incidence of neoplasms in a post mortem series, considers that the percentage of post mortems held on hospital deaths and the availability of beds for the chronic sick to take inoperable cases of malignant disease, affect the percentage of different neoplasms in any series. Also, when post mortems are performed infrequently they tend to include a high proportion of cases of undiagnosed neoplasms and of cases where symptoms were due to metastatic deposits.

The present post mortem series demonstrates the truth of Willis' statement and shows how not only the incidence of lung cancer but also the cell-type frequencies found in lung cancer in a post mortem series are dependent upon influences which are not entirely of a medical nature.

In the period of the survey post mortems were held on 43 per cent of hospital deaths, and the hospital record of death certificates shows that post mortems were held on 41 per cent of patients registered as dying from lung cancer.

Post mortem examination permission is requested by administrative staff, usually on the advice of the medical staff and also directly by the medical staff. When post mortems are held the death certificates are usually completed after the post mortem result is known. Facilities do exist at the hospital for transferring at least some cases of inoperable carcinomas to hospitals for the chronic sick.

These factors mean that there is some ante mortem selection of cases, by transfer of diagnosed inoperable patients ; and also considerable selection later, due to the method of asking for post mortems and their frequency. There is a far higher proportion of cases of undiagnosed and rare disease in the post mortem series than in the total hospital deaths.

*Age distribution of patients* (Fig. 6)

As in most reported post mortem series, the patients were considerably older than in clinical series.

The clinical course of untreated lung cancer is usually only a matter of months, so the different ages of the biopsy and post mortem series cannot be explained by the latter patients being in a more advanced stage of the disease.

This older age distribution, like the sex distribution, is probably due to social and economic factors.

*Duration of stay in hospital*

In the 107 cases where the time spent in hospital was known from the records, 32 per cent were dead within a week of admission and 81 per cent were dead within a month. This is similar to the cases reported by Budinger (1958) where 20 per cent were dead within a week of admission and 80 per cent died within three weeks of admission.

The biopsy series has shown that among 419 untreated and mainly inoperable cases of lung cancer only 22 per cent died within a month. Comparison of the survival rates of these two series suggests that very few hospital beds can have been used as " chronic sick " beds, and that the cases in the post mortem series were very different clinically from the inoperable cases in the biopsy series.

FIG. 6.—Age distribution of post mortem series patients..

*Symptoms causing admission to hospital* (Table VI)

In only 30 per cent of cases were the symptoms which caused the admission of the patients referable to the respiratory system. In 43 per cent of cases the main symptoms were caused by distant metastases.

TABLE VI.—*Systems Giving Rise to Symptoms which lead to Admission of Post Mortem Series Patients*

| | | |
|---|---|---|
| *Medical* | | |
| Respiratory system (primary lesions) | 48 | |
| Central nervous system (cerebral metastases cerebral thrombosis, peripheral neuropathies) | 14 | 86 |
| Cardiovascular system (coronory thrombosis, pericardial involvement) | 8 | |
| Haematological (thrombocytopenia, leucoerythroblastic anaemia) | 5 | |
| Vague (anorexia, asthenia, loss of weight etc.) | 11 | |
| *Surgical* | | |
| Gastrointestinal tract (liver, pancreas and peritoneal metastases and peptic ulcers) | 24 | 37 |
| Orthopaedic (fractures, rheumatism, osteodystrophy) | 7 | |
| Urinary (renal secondaries, unrelated prostatism) | 6 | |

In 30 per cent of cases these symptoms were mainly "surgical" and at the time of death 18 per cent were still in surgical wards. In eight cases symptoms and death were due to apparently unrelated diseases (cerebral thrombosis 1, coronary thrombosis 1, bleeding or perforation of peptic ulcers 5).

The surprisingly high mortality from peptic ulcers in this series does not represent the whole incidence of peptic ulceration among the cases, as many ulcers must have been overlooked. A similar association was noted by Lea (1952) who found that 12·1 per cent of a necropsy series of lung cancer cases had peptic ulceration.

*Diagnostic accuracy*

The accuracy of clinical diagnosis has been assessed—from case records in 111 cases, and from post mortem report clinical abstracts in 12 cases (where the case records were missing at the time of review). Five cases who were brought to hospital dead or died in the Casualty Department have not been included. Case records of patients whom I thought had not been correctly diagnosed clinically have been assessed independently by a consultant physician to the hospital, who has always agreed with my own opinion.

A correct clinical diagnosis was made with 48 per cent of patients. No mention of lung cancer appears in 25 per cent of the case records. The remaining 27 per cent were difficult to assess — usually secondary carcinoma was diagnosed and the primary site was uncertain though the lung was considered a likely site. It is clear from the case notes that once a firm diagnosis of secondary malignant disease had been established the clinicians desisted from further investigations for humanitarian reasons.

Diagnostic accuracy was lower in women (28 per cent) than in men (52 per cent). To a considerable extent this was because the possibility of lung cancer is often overlooked with female patients, but also it was due to the higher proportion of women suffering from metastatic disease, which itself was due to the cell-type distribution in women.

Three cases were diagnosed wrongly at post mortem examination as pancreatic carcinomas, and the diagnoses were corrected after histological examination and re-examination of the lungs. All three had oat-cell carcinomas.

The diagnostic accuracy of this series (48 per cent) is considerably lower than the 61 per cent in cases reported by Willis (1960) and the 54 per cent reported by Bonser (1959). The explanation is probably that the post mortem rate at Broadgreen Hospital was 17 per cent lower than in Willis' series, and naturally included a higher proportion of undiagnosed cases. If it is assumed that hospital deaths registered as due to lung cancer but without post mortem examination were correctly diagnosed, the diagnostic accuracy in the ten year period would be 77 per cent. This figure is probably close to the truth in a hospital which by the proximity of the Thoracic Centre is unusually conscious of lung cancer.

### *Histological Features* (Table VII)

*Specificity of cell-types*

Blocks of tumour from the primary site, regional lymph nodes, local spread and distant metastases were examined. 513 blocks of tumour were taken from 128

cases, an average of five blocks in all cases where the growth had extended beyond the primary site. All sections have been re-examined.

In 119 cases the cell-type remained constant throughout the tumour and its metastases. In nine cases (7 per cent) variations in cell-type were seen, and usually when these were present, mixtures of at least three cell-types were found.

*Cell-type characteristics*

Squamous carcinoma occurred infrequently, but these cases were the most accurately diagnosed cancers in the series. Only a third of the patients showed any metastic tumours and death was usually due to septic lung complications or unrelated conditions.

Oat-cell carcinoma was the commonest form of tumour with the highest incidence of metastases and, probably for this reason, was the least accurately diagnosed.

Carcinoma simplex was slightly less common than squamous carcinoma, but was diagnosed correctly almost as often. Metastases were more frequently seen than were the squamous carcinomas, but less so than in the other types.

Adenocarcinoma was the second commonest tumour, and a high proportion of them had metastases. Diagnostic accuracy with these tumours was low.

### MASS X-RAY CAMPAIGN, LIVERPOOL 1959

In February and March 1959 a Mass X-ray Campaign was held in Liverpool, in which 454,286 people were X-rayed, representing 76·5 per cent of the population over the age of 15 years. The surgical aspects of this campaign have been reported by Waddington (1960).

118 proved cases of lung cancer were discovered, and in 101 cases the histological type of tumour was reported from biopsy or resection specimens.

As such a campaign provides another cross-section of lung cancer patients all available tumours have been re-examined and re-typed where necessary. Ninety-two cases were diagnosed or operated on in Broadgreen Hospital or Aintree Hospital and all these sections were available. There is, therefore, some overlap of this series with the biopsy and operation series. Dr. E. Mavis McConnell of Liverpool Chest Hospital and Dr. H. Vickers of Walton Hospital each gave biopsy specimens from cases where no resection was undertaken.

The cell-type distribution of these 95 cases is shown in Table VII.

TABLE VII.—*MMR. Survey—Liverpool* 1959

| | | |
|---|---|---|
| Squamous carcinoma | 40 | 42 per cent |
| Oat-cell carcinoma | 16 | 16·5 per cent |
| Carcinoma simplex | 17 | 17·5 per cent |
| Adenocarcinoma | 14 | 14·5 per cent |
| Adenomatosis | 1 | 1 per cent |
| Carcinoma | 7 | 7 per cent |

### DISCUSSION

This study was undertaken in an attempt to find answers to four questions, which were :—Is lung cancer so pleomorphic that histological classification is valueless ? If not, do the cell-types of lung cancer have characteristic biological properties ? What is the cause of the differing cell-type frequencies reported in many series ? What is the true cell-type distribution of lung cancer in a population ? It is thought that satisfactory answers can now be given to the first three questions, but that there is no practical way of finding the answer to the last question.

Apart from these problems, the study has produced evidence which calls for modification of an accepted method of assessing histological alterations in the incidence of lung cancer in a population (Kreyberg, 1959), and other evidence which conflicts with arguments still used in support of the view that there may not be any increase in the incidence of lung cancer (Willis 1960). Both these points will be discussed.

*Mixed-cell Types*

Their frequency in the bronchial biopsy carcinomas was only 1·5 per cent, but because of the small amount of tissue available in each case little can be deduced from this series. In the operation specimens the incidence of mixed types was 3 per cent, and as an average of four blocks of tumours was examined in each case, as well as any lymph nodes attached to the specimens, this figure is of considerable value. The highest frequency of mixed types, 5·5 per cent, was found in the post mortem cases, in which an average of five tumour blocks was examined in cases where metastases were present, the blocks including tissues from the metastases.

A higher proportion of mixed types was found in cases where more blocks were examined, but I do not think that this process could be extended further, or that if ten blocks per case has been studied the incidence of mixed types would have become very much higher.

Half the mixed tumours contained mixtures of all cell-types, and often these mixed patterns were found in all sites and metastases examined. The other half of the mixed tumours were roughly equal numbers of squam-adenocarcinomas, squam-oat-cell carcinomas and adeno-oat-cell carcinomas.

If squamous metaplasia on the surfaces of oat-cell tumours and adenocarcinomas had been classified as mixed tumours, and if a glandular pattern in parts of oat-cell carcinomas had been called adeno-oat-cell carcinoma, the frequency of mixed cell-types might have been about 20 per cent of lung cancers, which is near the 23 per cent quoted by Willis (1960). However, I agree with Walter and Pryce (1955) that these features do not represent mixed forms of lung cancer.

*Biological Features of the Cell-types*

Even though consistency of cell-type has been found in lung cancer it does not follow that cell typing is of value unless it can be shown that individual cell-types have characteristic biological and prognostic qualities.

Many attempts have been made at grading other malignant tumours, mainly based upon degrees of differentiation and rate of cell division, but in lung cancer the problem is more difficult as it involves assessing the significance of several quite different histological pictures and attempting to show the inter-relationship of tumours of diverse appearance. Many arrangements of the cell-types, or spectra of lung cancer, have been made, based upon different factors such as histological similarity, supposed site of origin, relative malignancy or theories of aetiology.

In the present study the existence of mixed cell-type tumours has shown that the final entity is lung cancer ; but also it has been possible to arrange the cell-types in an order or gradation according to the features, which have been described in this survey and are summarised in Table VIII. This order is—squamous carcinoma, carcinoma simplex, adenocarcinoma, oat-cell carcinoma ; and the biological features which fit into this arrangement are the sex ratio, age distribution, size of tumour and regional lymph node involvement, and frequency of metastases.

TABLE VIII.—*Cell Type Characteristics from all Series of Lung Cancer*

| | Squamous carcinoma | Carcinoma simplex | Adeno-carcinoma | Oat-cell carcinoma |
|---|---|---|---|---|
| SEX—m/f ratio in biopsy, operation specimen and post mortem specimen series | 22:1, 23:1, 17 : 1 | 17:1, 14:1, 6 : 1 | 5·9:1, 5·4:1, 3·3 : 1 | 4·1:1, 3·3:1, 3 : 1 |
| AGE—percentage under 50 years age at tine of diagnosis | 17 (male) 21 (female) | 17 | 20 | 35 (male) 43 (female) |
| SIZE OF PRIMARY TUMOUR—percentage of tumours in operation series under 40 mm. diameter | 67 | 60 | 58 | 55 |
| LYMPHATIC SPREAD—percentage of large tumours in operation series with lymph nodes involved | 35 | 42 | 50 | 66 |
| BLOOD-BORNE METASTASES—percentage in post mortem series | 33 | 60 | 65 | 81 |
| SURVIVAL—percentage alive in biopsy series cases three months and six months after diagnosis | 56 35 | — | — | 38 12 |
| DIAGNOSTIC ACCURACY—percentage correctly diagnosed in post mortem series | 64 | 60 | 41 | 35 |

The table shows that carcinoma simplex has features which are very similar to those of squamous carcinoma and that in many ways oat-cell carcinoma is similar to adenocarcinoma, particularly regarding sex distribution, lymphatic spread and blood-borne metastases. This pairing of squamous carcinoma with carcinoma simplex, and adenocarcinoma with oat-cell carcinoma has not been based upon any histological similarity but solely on the other findings of the survey.

Histologically it is easy to accept that there could be a close link between squamous carcinoma and carcinoma simplex by assuming that the latter are all poorly differentiated squamous tumours. Both types usually arise from the larger bronchi. It is not so easy to accept a link between adenocarcinoma and oat-cell carcinoma, in spite of the acinar structure of some oat-cell tumours. Oat-cell tumours are usually central growths and many have a demonstrable bronchial site of origin, while adenocarcinomas are peripherally placed and detailed examination nearly always fails to show any bronchial origin. The nuclei of these two types of tumour are usually quite different. Another interesting but little recorded feature of oat-cell tumours is the occurrence of pools and strands of haemotoxophilic Feulgen-positive material in necrotic parts of many tumours, particularly in the walls of small vessels, which has been described by Assopardi (1959). This curious phenomenon was seen in many oat-cell carcinomas in the present study, but never in adenocarcinoma or in any other form of lung cancer.

### *Explanation of Variable Cell-type Frequencies in Different Series*

This general question can be answered by referring to the present series, which are typical of many other which have been recorded.

*Biopsy series*

The bronchial biopsy carcinomas reflect the true cell-type distribution of cancers which arise in the larger bronchi, are within reach of the bronchoscope and usually produce early symptoms. Squamous carcinomas were the commonest types but oat-cell tumours were present in a third of the cases, while adenocarcinomas were rarely found.

The only selection in this series arose from the site of the tumour and perhaps the age of the patients, because if bronchoscopies had been performed on older patients probably the frequency of squamous carcinoma would have been even higher.

There are few accounts of purely bronchial biopsy lung cancers for comparison with the present cases.

*Operation specimen series*

Though these cases are gathered in a wider diagnostic net than the biopsy series, they do not represent the true cell-type distribution of lung cancer. Selection of cases for operation is naturally influenced by the size of tumour, the degree of local spread and the presence of metastases. Choice of cases suitable for surgery is also influenced by the attitude of surgeons to different cell-types of cancer, which often are known before operation is decided upon.

As squamous carcinoma remains localised longer than do other forms, and as some surgeons are prepared to attempt radical surgery on borderline carcinomas if they are squamous tumours but not if they are oat-cell carcinomas, the frequency of squamous tumours is always high and that of oat-cell tumours low in any operation series compared with a biopsy series, and the cell-type frequencies are further altered by the inclusion of peripheral adenocarcinomas.

*Post mortem series*

This is a highly selected series and though by many considered to be representative of the overall cell-type distribution I regard post mortem studies as the least reliable method of assessing the frequency of lung cancer, or of its cell-types, in a population.

As most acute general hospitals try not to keep in their wards cases of diagnosed inoperable lung cancer these victims mostly die in " chronic sick " hospitals or in their homes. When diagnosed cases die in hospital they frequently escape post mortem examination because, the diagnosis being known and the condition common, they are not of much general interest. For these reasons the post mortem series contains a high proportion of patients who died shortly after admission (before there was time for diagnosis or transfer elsewhere), of patients who died from acute complications of lung cancer (such as pneumonia or pericarditis) and of patients who had symptoms solely from metastatic tumours (often with unknown primaries). Other ways in which the series is not typical of lung cancer are the sex and age distribution.

As squamous tumours are relatively easily diagnosed, produce symptoms early and metastasise late, they were uncommon in the post mortem series. When they occurred, admission had usually been due to acute complications such as pneumonia, or unrelated conditions like the complications of peptic ulceration.

On the other hand, oat-cell carcinoma and adenocarcinoma were common, as they were more difficult to diagnose, are relatively common in women, and often metastasise early and produce their main symptoms from the metastases.

*Mass radiography series*

This was a mixed series comprised of both biopsy and operation specimens with the latter providing the histological diagnosis in 68 per cent of cases. In the present series, as in most others, compared with an operation series, the frequency of squamous carcinoma was low, probably because patients with squamous carcinoma had had earlier symptoms and had attended hospital clinics rather than mass radiography centres. The incidence of adenocarcinoma was higher than in the operation series.

The observations based upon the present series appear to be true of most published studies. By looking at the cell-type frequencies in recorded series it is nearly always possible to anticipate the type of material that has been studied.

The difference in cell-type frequencies found in lung cancer collected from different types of material is so great that from an epidemiological angle it is useless to report a series where the source of the material is not precisely defined, and it is only of limited value to report cases from mixed sources unless the cases from each source are analysed separately.

### *Cell Typing as an Indicator of Lung Cancer Frequency*

The increased incidence of lung cancer over the last half century is mainly attributed to cigarette smoking and atmospheric pollution. In 1954 Kreyberg divided lung cancers into two groups which he thought were aetiologically distinct. His Group I contained squamous, large cell and small cell carcinoma, which he considered to be caused by external factors such as cigarette smoking and atmospheric pollution. Adenocarcinomas, adenomatosis and bronchial adenomas made up his Group II, which he found were more equally distributed between the sexes and thought were unrelated to external factors.

This view was supported by Doll, Hill and Kreyberg in 1957, when they showed that there is a close relationship between the amount of tobacco smoked and the development of squamous, large cell, and small cell carcinoma but no such correlation existed with the Group II tumours. In 1959 Kreyberg omitted large cell carcinoma from his Group I tumours, but showed that these tumours are also related to other external factors, such as nickel and asbestos. He put forward the thesis that the ratio between Group I and Group II tumours in men in any consecutive unselected material provides information as to the frequency of lung cancer in a population, and that an alteration in this ratio will indicate a change in the lung cancer situation. In support of this theory he quoted Norwegian figures, where the ratio before the increase of lung cancer was 1·8 : 1, and later became 38 : 1. However, a later study (Ferrari and Kreyberg, 1960) of 78 cases in Venice, where lung cancer is prevalent, showed a ratio of 3·2 : 1, not much different from Norway where lung cancer has a low incidence.

I think that Kreyberg's basic conception of a division of lung cancer into two aetiologically distinct groups is an approach that may be of great value, and probably carcinoma simplex (his large cell carcinoma) should appear in Group I, as it behaves so like squamous carcinoma. However, his method of comparing group ratios in different series and deducing therefrom lung cancer incidence changes needs closer examination and some standardisation of studied series is essential.

The present survey has shown that the cell-type frequencies and, therefore, the Group I : II ratio, varies according to the type of material being studied. Liverpool is certainly a city with a very high incidence of lung cancer, but the Group I : II ratio in men (confining Group I to squamous and oat-cell tumours) was 17·8 : 1 in the biopsy series, 5·04 : 1 in the operation specimens and only 1·84 : 1 in the post mortem cases. These three ratios were obtained from studies of the same population over a constant period, and according to Kreyberg's theory show that in the same period Liverpool had about three times as much, and only two thirds of, the lung cancer incidence of Norway.

It is obvious that all bronchial biopsy series will have a very high ratio, for example, the cases typed by Doll, Hill and Kreyberg (1957), which one can deduce were mainly diagnosed from bronchial biopsies, have a ratio of 20·8 : 1. All post mortem series will have a low ratio because of the high proportion of adenocarcinomas and low proportion of squamous tumours ; and operation series will occupy an intermediate position.

In his 1959 paper Kreyberg illustrates the value of using his ratio for assessing increases in lung cancer by comparing Norweigian figures up to 1946 with those after 1948, which showed that the ratio had doubled. Unfortunately the value of this example is reduced by the knowledge that the earlier ratio was obtained from post mortem material, while the later ratio was from mainly clinical material. In spite of these obvious defects which may have lead to a false conclusion, I think that a study of the Group I : II ratio in large and strictly comparable materials may provide an accurate index of any increase of lung cancer caused by cigarette smoking or atmospheric pollution.

*The Increase in Lung Cancer*

One other aspect of lung cancer deserving comment is its increased incidence in recent years. In the ten year period of this study the number of cases of lung cancer registered with the Liverpool Cancer Control Organisation more than doubled, the number of positive carcinoma bronchial biopsies nearly doubled (allowing for those seen at new clinics), the number of operation specimens rose steeply and steadily, and there was a small increase in the post mortem material.

In spite of this clinically-apparent increase there are still those who claim that there is as yet no proof of an increase in lung cancer, but that an apparent increase may be due to better diagnosis, unproved diagnosis, or an ageing population. One of the common arguments used against there being much increase is based upon post mortem statistics (Willis, 1960).

Though an increase has been apparent clinically, and in the Registrar General's returns since the first World War, the percentage of lung cancer cases in post mortem series up to 1930 remained constant. Since then there has been an increase of about 50 per cent in the post mortem figures. Willis points out that in the period when there was a ninefold increase in death certificates from lung cancer the increase in the post mortem rate was only 50 per cent.

Nearly all these statistical studies have been made in teaching hospitals, where the post mortem rate is not always very high and where there are few cases of diagnosed inoperable malignant disease. The two factors which mainly influenced the frequency of lung cancer in the post mortem series at Broadgreen Hospital were the transfer of cases to hospitals for the chronic sick and the post mortem rate. The same factors would influence a similar series in a teaching hospital where, though the post mortem rate might be higher than at Broadgreen Hospital, there would be non-admission of cases known to be inoperable, and more transfer of cases found to be inoperable. Also as post mortem series which are not 100 per cent of deaths contain a higher proportion of undiagnosed cases than do the hospital deaths, and as bronchoscopy is now a common investigation making lung cancer more easily diagnosable, a smaller proportion of lung cancer cases now occurs in nearly all post mortem series. These factors clearly affected the lung cancer incidence in the present post mortem series, but they are often overlooked by those who study post mortem statistics and draw conclusions based purely on mathematics.

### SUMMARY

An analysis of the histopathology of over 1900 cases of lung cancer occurring in the Liverpool area in the decade 1950–1960, showed that in no more than 6 per cent of cases was a mixture of cell-types found in the tumours. The rest of the lung cancers were classified as squamous carcinomas, carcinoma simplex, oat-cell carcinoma, adenocarcinoma and adenomatosis.

A study of the clinical records at a follow-up of patients showed that cell-types possessed distinctive biological properties, regarding age and sex distribution, size of tumours, lymphatic spread extent of metastases and survival. In order of increasing malignancy the tumours were: squamous carcinoma, carcinoma simplex, adenocarcinoma and oat-cell carcinoma.

The lung cancers were studied in three main series, in which the histological diagnosis had been made from specimens obtained at biopsises, operation and post mortem. The cell-type frequencies found in the series showed marked differences, which could be explained as being due to the biological properties of the cell-types and to external factors influencing the selection of cases in each series. None of the series reflects the cell-type frequency in a population.

The method of using the Group I tumour : Group II tumour ratio as in indication of the frequency of lung cancer in a population is only of value if strictly comparable series are studied.

I wish to thank the Medical Staffs of the Regional Thoracic Service and of Broadgreen Hospital, for the use of their patients' case records; Dr. L. Findlay for his assessment of clinical diagnostic accuracy of the post mortem series patients; Dr. E. Mavis McConnell, Pathologist to the Liverpool Cancer Control Organisation, for much assistance in tracing patients' records; and Dr. Rachel M. Rawcliffe for reading and correcting the manuscript.

Especially do I wish to acknowledge my debt to Mr. A. Robertson, F.I.M.L.T., Senior Histology Technician, not only for all the histological preparations which I have examined, but also for his tireless assistance in finding old sections and in abstracting and tabulating old reports, without which help the work could not have been possible.

### REFERENCES

ANDERSON, W. A. D.—(1948) ' Pathology '. London (Henry Kimpton), p. 745.
AZZOPARDI, J. G.—(1959) *J. Path. Bact.*, **78**, 513.
BARNARD, W. G.—(1926) *Ibid.*, **29**, 241.
BIGNALL, J. R.—(1958) In ' Carcinoma of the Lung '. Ed. Bignal, J. R. London (Livingstone), pp. 168, 184, 185.
*Idem* AND MOON, A. J.—(1955) *Thorax*, **10**, 183.
BONSER, G. M.—(1959) *Brit. J. Cancer*, **13**, 1.
BUDINGER, J. M.—(1958) *Cancer*, **11**, 106.
CHRISTIANSEN, T.—(1953) *Brit. J. Cancer*, **7**, 428.
DOLL, R. AND HILL, A. B.—(1952) *Brit. med. J.*, ii, 1271.
*Iidem* AND KREYBERG, L.—(1957) *Brit. J. Cancer*, **11**, 43.
FERRARI, E. AND KREYBERG, L.—(1960) *Ibid.*, **14**, 609.
GALLUZZI, S. AND PAYNE, P. M. (1955) *Ibid.*, **9**, 511.
GIFFORD, J. H. AND WADDINGTON, J. K. B.—(1957) *Brit. med. J.*, i, 723.
HINSON, K. F. W.—(1958) in ' Carcinoma of the Lung '. Ed. Bignall, J. R. London (Livingstone), pp. 115, 133.
JAKOBSEN, A.—(1953) *Brit. J. Cancer*, **7**, 423.
KREYBERG, L.—(1954) *Ibid.*, **8**, 199.—(1959) *Acta. Un. int. Cancr.*, **15**, 78.
LEA, A. J.—(1952) *Thorax*, **7**, 305.
LIEBOW, A. A.—(1952) ' Tumours of the Lower Respiratory Tract. Atlas of Tumour Pathology ". Sec. 5. Fasc. 17. Washington, D.C. (Armed Forces Institute of Pathology), p. 67.
McBURNEY, R. P., McDONALD, J. R. AND CLAGETT, O. T.—(1951) *J. thorac. Surg.*, **22**, 63.
MASON, G. A.—(1949) *Lancet*, ii, 587.—(1960) *Thorax*, **15**, 1.
MULLANEY, P. J.—(1958) *Brit. J. Cancer*, **12**, 327.
NICHOLSON, F., FOX, M. AND GRAHAM BRYCE, A.—(1957) *Lancet*, i, 296.
PHILLIPS, F. J., BASINGER, C. E. AND ADAMS, W. E.—(1950) *J. thorac. Surg.*, **19**, 680.
RAEBURN, C. AND WALTER, J. B.—(1956) *Lancet*, i, 778.
SELLORS, T. H.—(1955) *Brit. med. J.*, i, 445.
STEINER, P. E.—(1953) *Acta Un. int. Cancr.*, **9**, 42.
UMIKER, W. AND FRENCH, A. J.—(1960) *Cancer*, **13**, 1053.
WADDINGTON, J. K. B.—(1960) *Med. Offr*, **104**, 293.
WALTER, J. B. AND PRYCE, D. M.—(1955) *Thorax*, **10**, 107, 117.
WHITWELL, F.—(1961) *Brit. J. Cancer*, **15**, 429.
WILLIS, R. A.—(1960) ' Pathology of Tumours '. 3rd edition. London (Butterworth), pp. 130, 77, 72, 360, 369.